# Social Media Improves Students’ Academic Performance: Exploring the Role of Social Media Adoption in the Open Learning Environment among International Medical Students in China

**DOI:** 10.3390/healthcare9101272

**Published:** 2021-09-26

**Authors:** Muhammad Azeem Ashraf, Muhammad Naeem Khan, Sohail Raza Chohan, Maqbool Khan, Wajid Rafique, Muhammad Fahad Farid, Asad Ullah Khan

**Affiliations:** 1 Research Institute of Educational Science, Hunan University, Changsha 410082, China; azeem@hnu.edu.cn; 2 School of Social and Behavioral Sciences, Nanjing University, Nanjing 210023, China; muhammadfahad@smail.nju.edu.cn; 3 School of Information Management, Nanjing University, Nanjing 210023, China; sohail@smail.nju.edu.cn (S.R.C.); DG1714502@smail.nju.edu.cn (A.U.K.); 4 Department of Information Sciences, University of Education, Lahore 54770, Pakistan; 5 Department of IT and Computer Science, Pak-Austria Fachhochschule Institute of Applied Sciences and Technology, Haripur 22621, Pakistan; maqbool.khan@fecid.paf-iast.edu.pk; 6 Department of Computer Science and Operational Research, University of Montreal, Montreal, QC H3C 3J7, Canada; wajid.rafique@umontreal.ca

**Keywords:** open learning, engagement, collaboration, communication, electronic-learning

## Abstract

Numerous studies have examined the role of social media as an open-learning (OL) tool in the field of education, but the empirical evidence necessary to validate such OL tools is scant, specifically in terms of student academic performance (AP). In today’s digital age, social media platforms are most popular among the student community, and they provide opportunities for OL where they can easily communicate, interact, and collaborate with each other. The authors of this study aimed to minimize the literature gap among student communities who adopt social media for OL, which has positive impacts on their AP in Chinese higher education. We adopted social constructivism theory (SCT) and the technology acceptance model (TAM) to formulate a conceptual framework. Primary data containing 233 questionnaires of international medical students in China were collected in January 2021 through the survey method. The gathered data were analyzed through structural equation modeling techniques with SmartPLS 3. The results revealed that perceived usefulness, perceived ease of use, and interactions with peers have positive and significant influence on OL. In addition, OL was found to have positive and significant influence on students’ AP and engagement. Lastly, engagement showed a positive impact on students’ AP. Thus, this study shows that social media serves as a dynamic tool to expedite the development of OL settings by encouraging collaboration, group discussion, and the exchange of ideas between students that reinforce their learning behavior and performance.

## 1. Introduction

The term social media (SM) is considered as a form of communication through electronic platforms, which intends to make online communities for users to share knowledge, information, opinions, messages, and other content [1]. In the 21st century, SM became an essential part of human life, while the use of SM has spread across the world. In 2020, almost 3.06 billion individuals from all walks of life used at least one SM platform, such as WeChat, Facebook, Twitter, Weibo, WhatsApp, and Instagram, in their daily life [2]. The use of SM has become an integral part of intellectual work, and students posting study-related material on SM platforms is considered a reliable source of information that is important to each community, such as those of students, customers, and employees [3]. The users of SM (computational technology that helps to develop and share ideas, perceptual knowledge, professional interests, information, and other expressions through social network platforms) may read or see their friends’ activities online without direct contact with them [4]. Furthermore, SM networking sites utilize features, such as comments, postings, digital photographs, video-sharing, and data about online interactions, that provide vitality for SM users [4]. People who use SM are called netizens. Netizens often access online platforms using the internet or other web technologies on their computers or laptops, or they download programs to the mobile devices (such as smartphones or tablets) that expand the functionality of SM networks [4]. The use of SM platforms in educational activities is increasing day by day. Because of the engagement of SM users with such services, they usually develop highly interactive platforms wherein students may create or exchange ideas and discuss information or previously published online content in user-created groups. SM promotes interactions between teachers, subject specialists, students, communities, and major companies. This revolution is the focus of new and creative information technology (IT) areas [3].

SM has been used in medicine extensively, as almost one-third of the adults with internet access have viewed different social media sites concerning the medical experience of other people, while almost 6% of these people have participated through text messages, comments, replies, photos, recorded files, and personal assessments of health conditions by professionals [5]. SM has provided opportunity for individuals with specific illnesses to take part in online communities to share their personal experiences, contact other people to learn from their experiences, and contact medical specialists to glean comprehensive knowledge about their illnesses. Similarly, healthcare workers including doctors and nurses are also using SM significantly in their professional lives, where they exchange information regarding their professional problems as well as clinical experiences [6]. Likewise, current medical students are also using SM broadly as a tool of communication among their educational and professional lives. In medical education literature, communication, peer feedback, collaboration, material sharing, and social media ability are reflected as the major aspects essential for SM usage among medical students [7]. Since SM holds massive importance in educational settings, Davis, Ho, and Last suggested that medical schools revise their syllabi by integrating social media in their instruction in ways that are innovative, timely, and evidence-based to meet the demands of this dynamic learning landscape [8]. Thus, studies on the role of SM use in medical education would enhance and improve the teaching and learning environments for both medical students and medical practitioners [3].

In addition, SM (characterized by user-generated content (UGC)) enables students “to create, circulate, share, and exchange information in a variety of formats and with multiple communities” [9]. WhatsApp, WeChat, Facebook, Instagram, Pinterest, Linked In, Snap Chat, Twitter, Telegram, Baidu, Google+, SlideShare, Weibo, Tumblr, and related websites are the most popular platforms among SM users [1]. Google+, which provides a single destination to students to easily and quickly communicate and discuss their problems, is widely used all around the world. WeChat is widely used by people in China for social networking [1]. Thus, social media has now become a popular platform for knowledge sharing between medical students and teachers [7]. SM platforms have enabled students to work together, interact with colleagues and classmates, and acquire the latest knowledge, which has positive impacts on their AP [10]. One constructive effect of using SM platforms is the introduction of the public to consumer data, ideas, and programming, which has promoted further technical advances and increased knowledge in educational institutions [9].

OL is a terminology that indicates that “an inner feeling conveyed in this technique through external actions involving students in existing, continuous learning groups or teams” [11]. Rapid expansions of information communication technology (ICT) have led to pragmatic practices. Many terms such as online learning, blended learning, web-based learning, m-learning, and computer-mediated learning have been used in the literature to show the importance of technology in academic learning. All these terms have distinct features, but they are linked to each other through the ability to use a computer that is connected to a network, which provides the opportunity to study from any place at any time [6,11]. OL can be characterized as an instrument that has made knowledge-learning practices more innovative, student-centered, and flexible [4]. OL is a procedure of reciprocity, communication, and collaboration within student communities in which students share their difficulties with other group members and receive solutions, guidance, and advice; it also improves their learning processes, enhances abilities such as collaboration and social abidance, and creates productive interplay as a potential tool for learning [11]. Additionally, OL makes it easier to elaborate and develop critical thinking, materials interchange, and proficient knowledge on online platforms [12]. SM has become the essential tool for OL in student communities and others [3], and SM use is widely used as the main communication platform for student learning [11] because some of its associated tools are not too costly to enable their utilization and growth in acceptable and satisfactory settings for OL. SM has led to the wide distribution of several group exercises, such as sharing knowledge and information, communications, and interactions, in education, thus enhancing students’ learning potential.

Several scholars have examined the link between SM and AP, and they have highlighted many mixed results when using such platforms. For example, according to Ktoridou and Eteokleous [13], SM platforms allow students to interact with group members to find help in solving learning problems. Moreover, using SM platforms may enhance learning achievement in OL environments [11]; however, some studies have shown that students’ use of SM platforms for study (assignment) does not improve learning outcomes [14]. Hence, students must monitor and analyze the patterns of collaboration that emerge throughout OL on SM, where motivating cognitive skills, reflection, and metacognition is crucial for learning [11]. Nevertheless, earlier research revealed that students have negative attitudes regarding social media, as they believe that most SM platforms do not help them achieve AP [15,16]. According to Anderson and Jiang, the use and availability of SM platforms have led to a decline in AP [17]. However, other studies have found that there is no link between SM use and AP [18].

Alenazy, Mugahed Al-Rahmi, and Khan explained that students are suspicious of the idea that using SM platforms can aid them in measuring education sustainability [19]. Other scholars have claimed that while students prefer face-to-face contact with peers and lecturers, they have a favorable attitude toward learning activities integrated with SM platforms [20]. Therefore, more research is required in the field of attitude regarding SM platform use for OL and AP [11]. Cyberstalking and cyberbullying via SM platforms have been linked to psychological and emotional issues such as discomfort, anxiety, and insecurity [21,22]. However, the better integration of SM in academic courses has provided positive effects on students’ AP, such as improving motivation in learning and encouraging students to communicate with their teachers [20]. 

Despite having reached many countries, there remains a scarcity of studies on the use of SM platforms in higher education, especially in China. Thus, the authors of the current study sought to fill in this literature gap by investigating the use of SM platforms to achieve the goal of OL, positively affecting AP, and positively affecting student engagement (ENG). Following the literature gap, our study’s main objectives were:

1. To explore the factors that influence the use of SM platforms among international medical students throughout their studies.
2. To explore the effect of SM-based OL that promotes student AP.
3. To explore how medical students use SM to maintain their ENG with peers and their performance.

This research aimed to provide new opportunities to include SM platforms in progressive education in medicine, and to take advantage of the exciting benefits of OL tools in medical training. The present research model was based on two theories: SCT by Vygotsky [23] and the TAM by Davis [24]. The TAM is known as one of the most widely used models for analyzing attitudes about the use of SM platform technology, and SCT addresses interactions and their effect on the OL and ENG of students. These two theories were utilized to assess students’ AP, which is still seriously unexplored. Furthermore, there is a lack of research models for OL, AP, and ENG, including the use of SM platforms in the context of higher education in China. Hence, the goal of this research was to fill in the gaps in the literature by examining SM platforms’ characteristics utilized for OL and ENG that affect students’ AP.

## 2. Literature Review

Through the alteration of our social standards, values, and culture, SM has progressively become an important part of human society [25]. Information and content dissemination are becoming significant for people. The learning processes at education institutions have transformed the lives of individuals, including university students and (especially) women, by changing method of communication and engagement in learning [26]. These new media platforms play essential roles in the exchange of material between university students and society. Students now have the opportunity to share their routine life through photographs, comments, and the dissemination of ideas in social and academic discussions [27,28], and SM affects the everyday life of young people and especially university students [29]. Digital and social networking have revolutionized daily ways of communication by developing content, exchanging information, and consuming information [30].

SM platforms allow for social interaction and communication between users by exchanging knowledge and transforming monologues into dialogues between consumers [31]. SM, based on a specific philosophical worldview and technological underpinnings and functionalities, encompasses numerous internet-based tools and apps [32] that have enabled its users to distribute material across digital media and internet spaces [33]. It has provided chances for the inexpensive and viable online advertising of goods and services, it offers new ways of dealing with and coordinating interactions amongst users [34], and many SM users consistently disseminate and share their articles, images, videos, and records on different SM apps [35].

SM offers venues for students and the public to exchange ideas and information by discussing information with each other, as well as to build up relationships through social networking [13,36]. In today’s society, SM platforms and education are inextricably linked [37] because they work as central spaces for debate, discussion, and feedback among students and teachers [38]. SM platforms can be a valuable tool to enhance learning behavior [39] by allowing people to organize content; share information, movies, photos, communication, and coordination; and build social links with others based on collaborative efforts [13,40]. SM platforms include websites, wireless internet connections, and video or photo-sharing sites. At the moment, it is not just advantageous to participate in digital media sharing and social networking—it also enables social contact and communication through the development of brands and professional possibilities [41,42]. According to Wodzicki, Schwämmlein, and Moskaliuk, social networking offers a variety of resources that may be used for instant access to learning and information [43]. For instance, students of higher academic levels extensively use SM platforms for educational purposes [13]. In addition, these platforms have several other uses, such as entertainment and interactions with others [44].

Joachim, Geert, and Soetaert stated that the trustworthiness of these webpages is typically based on demonstrated taste and expertise, rather than on the institution’s association and recognition [45]. According to academics, SM platforms comprise a technology that is used to facilitate social relationships, facilitate collaborations, and enable negotiations among large populations [46]. SM platforms have allowed for the promotion of personalized learning environments as an educational strategy for enhancing self-regulated learning [47]. According to educational experts, SM platforms provide the majority of the characteristics of an excellent educational technology in terms of peer reaction, scholar mentoring, and matching the social circumstances of electronic learning (e-learning) [29].

## 3. Research Model and Hypotheses

In the current research, we incorporated two core theories (TAM and SCT) to develop a conceptual model to attain the research objectives. Firstly, Davis conceived a TAM to regulate the causal relationships between the internal views, perspectives, and intentions of users to adopt computer technology [24]. Scholars have extensively used the TAM to study information systems (ISs) and computer technologies (CTs). For instance, Chandra applied the TAM to investigate the adoption of online auctions by users [48]. 

The SCT defines knowledge as constructed in a collaborative way within a social context. It considers learning as a condition wherein individuals construct their personal meaning from the content and materials presented to them, rather than simply memorizing the information [23]. In addition, SCT is based on the idea that learning can be enhanced and made to be more constructive within the orbit of social process in cognition groups. Moreover, knowledge is an ongoing process that needs improvements with time, and learning is best accomplished when it follows social perspective in effective and constructive process [49,50,51,52,53]. According to Bhattacharjee [54], the emergence of constructivism research in the recent era has enhanced the tools and focus of media technologies for the fast transfer of information and knowledge to the next generation. Similarly, as suggested by Ershler and Stabile, learning is a process that results in the transmission of culture, which may attract constructivists to reconsider the influence of social media on culture [55]. The recent emergence of social media has massively affected attitudes towards education by changing the landscape of information availability. 

**Figure 1:**
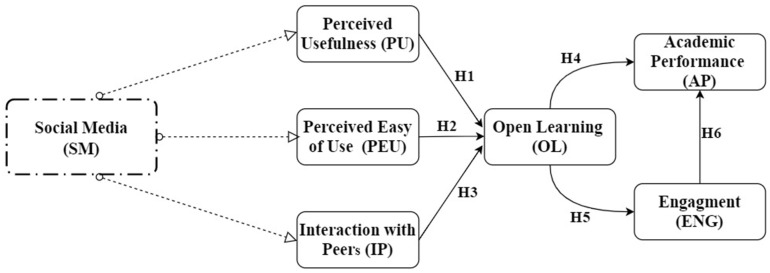
Conceptual model based on TAM and SCT.

In SCT, teaching and learning ought to focus on consuming content to develop means of understanding, and these contents have become abundant and easily reachable through social media. The effects of social media for SCT involves significant changes to the ways students often communicate, and how they acquire basic understandings. Thus, as social media permits the alteration, integration, and distribution of information, it has massive influence on the learning of individuals. The strengths of SM platforms follow the principles espoused by constructivists [56]. For instance, Churcher showed that SM platforms lead to online communities of learning practice [57]. Other studies have shown that SM platforms facilitate participation, communication, social interactions, the use of modern technologies, the use of online applications, collaboration, and the construction of personal meaning that satisfies the learning condition of constructivism [58,59]. Likewise, SCT suggests that information on OL activities, personal activities, and social interactions can be gathered through the use of modern tools of technology [60]. Figure 1 illustrates the conceptual model of this research.

### 3.1. Perceived Usefulness

PU refers the level to which a student thinks that using a specific technology would increase their job performance [61]. In our study, PU was defined as how much a user feels that SM platforms can be used for OL to enhance their AP. The current research provides evidence that PU affects the attitudes and intentions of those using technology [21,62]. Since PU has a direct impact on attitudes, it was assumed to have an indirect impact on intention to use technology. Hence, the following hypothesis was formulated:

**Hypothesis** **1.** *PU is positively related to OL.*

### 3.2. Perceived Ease of Use

PEU refers to the level a student perceives that the use of a specific technology is effortless [24]. In this research, PEU refers to the extent to which an individual believes that using SM platforms for OL will increase their AP. Al-Rahmi et al. [11] stated that PEU has significant impacts on e-learning acceptance and adoption. Several studies have shown that PEU affects PU, though both have positive impacts on the behavioral intention of adopting technological systems [63]. In addition, several studies have shown that the intention of continuing to use SM platforms for OL is largely influenced by PEU [11,19]. Accordingly, we formulated the following hypothesis:

**Hypothesis** **2.** *PEU is positively related to OL.*

### 3.3. Interact with Peers

SM platforms allow students to communicate, share content with classmates, and connect with others [64]. In today’s world, most students are regular users of SM platforms to remain aware of and updated on current events [65]. Utilizing SM platforms in academic-related activities such as discussions allows students to participate in subject discussions and interact with content [66]. This single destination of conversation paves the way for communication and enhances students’ learning strengths, which can move beyond the subject raised by teachers or hosts [67]. SM platforms are the best resource for improving communication, promoting positive learning attitudes, encouraging students to seriously consider learning and learning activities, and maximizing social capital through virtual communications. It has been noticed that students or scholars in online settings spend time on SM platforms to work through the learning process [68]. It is believed that the use of SM platforms in educational institutions enhances the level of interaction between instructors and students [69]. According to Alamri et al. [68], learning tools are just as essential as learning objectives because they encourage social interaction, entail interactive learning, and aid open learning. Thus, we proposed the following hypothesis:

**Hypothesis** **3.** *IP is positively related to OL.*

### 3.4. Open Learning

OL can be defined as a learning process in which an individual has opportunities to work in a team or group so that learning is fostered through interpersonal interaction, group collaboration, and active learning [68]. Dumford and Miller observed that OL and student ENG through the use of SM platforms have significant relationships with team member interactions [70]. Balakrishnan and Gan used an SM platform adoption model to investigate the various factors that affect students’ intentions to use SM for learning based on, for instance, the commitment, competitive, and autonomous styles [71]. In addition, according to a study by Ratneswary and Rasiah, the use of SM platforms improves OL and establishes a strong and engaging bond between students and teachers [72]. Thus, the authors of this study claim that OL improves student AP. Based on earlier studies, we posited the following hypotheses:

**Hypothesis** **4.** *OL is positively related to AP.*

**Hypothesis** **5.** *OL is positively related to ENG.*

### 3.5. Engagement 

In the context of SM platforms, ENG creates a learning atmosphere characterized by discussion and interaction among colleagues that foster closer collaboration and communication [73]. Furthermore, research has shown that the use of SM platforms leads to positive AP and ENG experiences [74]. SM platforms are seen as online learning tools that offer significant benefits for better results and experiences through cognitive participation and social ENG [68]. To this end, OL enables the expansion of ENG in curriculum activities and knowledge-sharing systems [75]. According to Blasco-Arcas et al., students learn more effectively when they participate in appropriate cognitive processes, so student ENG is a significant explanatory variable for academic performance. In addition, SM platforms enable students to engage in knowledge construction, which ultimately involves a higher level of perceived learning. When students are engaged with learning activities, their AP improves [68]. Following prior studies, we proposed the following hypothesis:

**Hypothesis** **6.** *ENG is positively related to AP.*

### 3.6. Academic Performance

This study applied the concept of academic performance as the achievement of educational objectives in terms of knowledge acquisition and skills development [68]. Social media refers to the electronic platforms which allow their users to interact with other user users to share information [76]. Previous studies have observed some forms of impact of SM on AP [18,47,68], but there is very little research on SM and AP in the Chinese context, particularly on international students. Therefore, this study aimed at finding the impact of SM on students AP in open learning environments through the SCT and TAM models. In this research, perceived usefulness (PU), perceived ease of use (PEU), and interactions with peers (IP) were independent variables, and OL was chosen as the mediator variable. The dependent variables were ENG and AP (Figure 1). 

## 4. Methodology

This study was part of a large project funded by the National Natural Science Foundation of China (grant no. 71950410624) to investigate the role of internet and technology in improving teaching and learning practices in Chinese higher education. As indicated in previous sections, social media holds great impact in all aspects of teaching and learning, including in the medical field [3,4,5,6,7]. It has been debated in terms of its use as a tool of communication among individuals, ease of use, improvement in learning, and better professional development. Considering these outcomes, more evidence on educational usage of social media has yet to arise to evaluate to what extent medical practitioners can yield educational benefits from these resources. Therefore, the purpose of the current study is to explore the role of social media use as a tool of OL among international medical students in China. The population of this study comprised 231 international undergraduate and graduate medical students between the ages of 20 and 40 from universities in the Jiangsu province of China. This study focused on SM as an OL tool; learning platforms other than SM were not included. Prior to conducting this research, we analyzed the complexity of the term social media, because it has been defined and used differently in the previous literature. We considered all web-based tools that allow users to create and exchange content and enable them to interact with other people, as explained by Miller et al. [76]. 

As the study was located in China, we considered the most commonly used SM platforms in China, such as WeChat, Weibo, QQ, Tencent Meeting, and others [77]. WeChat is considered a super version of Facebook and is the most popular social media platform among people in China, and it provides many different services such as instant personal and group messaging, sharing of information/videos/news through WeChat Moments, payment services, marketing services, and many other services all in one app [77]. 

All participants gave their informed consent before they participated in the study, which was conducted in accordance with the Declaration of Helsinki 1975, revised in 2013. The period of data collection was from January to March 2021. We investigated the driving factors behind SM platform adoption for OL and its impact on student AP. 

### 4.1. Constructs Development and Pilot Study

A structured online survey questionnaire was used to collect data because the online data collection method is considered appropriate, fast, inexpensive, and able to minimize incorrect data and incomplete responses [78,79], as well as suitable to overcome difficult physical access due to long travel times and/or COVID-19 [80]. The study constructs of PEU and PU were defined based on work by Davis [24], and OL, IP, ENG, and AP were defined and measured following the works of Al-Rahmi et al. [81] and Alamri et al. [68]. AP was measured through the students’ self-reporting on their academic performance in the 2020–2021 fall semester. Each construct (multiple items) was measured on a five-point Likert scale (i.e., from strongly disagree to strongly agree). At the start of the survey, all respondents were informed that their participation was voluntary and were provided a brief overview of the purpose of the study. The respondents were assured that their information would be kept strictly confidential and used for research purposes only. Before the actual study, a pilot test or pilot study was carried out with 33 respondents to ensure the legibility of the survey questionnaire [13]. Based on feedback, small changes, such as to questionnaire terminology, were made. Detailed information regarding constructs and item measurements are listed in the Appendix A.

### 4.2. Formal Survey

To test the hypotheses, we distributed a revised questionnaire (Appendix A) through WeChat, QQ, and email. Before filling out the questionnaire, the respondents were informed that this questionnaire was only for those who use SM platforms for educational purposes for at least two hours a day. We used two pieces of software for analysis: Jamovi for the organization of demographic data and SmartPLS 3 for the data analysis model.

### 4.3. Descriptive Analysis

The respondents’ demographic information is shown in Table 1. The authors received a total of 297 responses, and the final sample contained 233 respondents, which is valid for data analysis. In the dataset, N = 104 were female students and N = 129 were male students.

### 4.4. Common Method Variance

We applied Harman’s single-factor test to assess the potential for common method variance (CMV) in our data [82]. The results demonstrated that the first factor’s value was 37.97%, which was lower than the recommended minimum value of 50%. In the data, we found no common method bias and no CMV issue.

## 5. Data Analysis 

We used “structural equation modeling (SEM)” to test the research hypotheses (Figure 1) with SmartPLS 3 software. We divided the structural equation model into two stages. In the first stage, we analyzed the measurement model to test the reliability and validity of the data, and in the second stage, we analyzed the relationships hypothesized by the structural model. 

### 5.1. Measurement Model

The results of Table 2 demonstrate the constructs’ reliability and validity. The factor loadings, Cronbach’s alpha (CA), composite reliability (CR), and rho_A of each construct were found to be greater than the value of 0.70 recommended by Hair, Hollingsworth, Randolph, and Chong in all cases [83]. The values of average variance extracted (AVE) of all constructs were higher than the value of 0.5 suggested by Fornell and Larcker [84]. An appropriate discriminant validity (defined as the degree that one construct differs from another construct [85]) was achieved because all correlations between dimensions were less than the square root of the AVE [84] (Table 3) and the heterotrait–monotrait (HTMT) relationship of the correlations between two constructs was less than 0.9 [86] (Table 3). Lastly, we examined variance inflation factors (VIFs) to analyze collinearity problem; they were found to be lower than 5 [87,88], which indicated that common method variance was not an issue in this study, as shown in Table 2.

### 5.2. Structural Model

**Figure 2:**
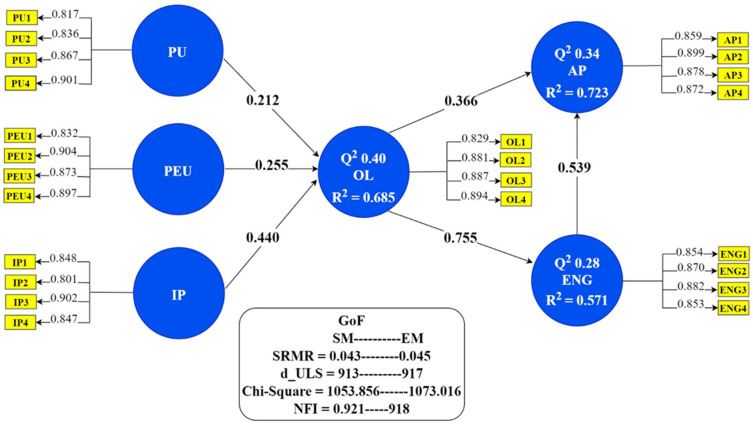
Results of proposed model.

To check the structural model, we examined the significant relationships among exogenous and endogenous variables. To examine the significance of the path coefficients, a bootstrapping procedure with 5000 resamples was performed [89]. Figure 2 illustrates the results of the structural model assessment, showing that all our hypotheses had significant relationships and that the overall model fit following bootstrapping allowed for significant values. Furthermore, Figure 2 shows that the three endogenous variables had substantial R^2^ values. The effect size (f^2^) of a structural model relationship measures the contribution of exogenous constructs in endogenous constructs. Following the work of Cohen [90], we found f^2^ values of PU -> OL 0.064, PEU -> OL 0.078, PE -> OL 0.194, OL -> SAT 1.329, OL -> AP 0.208, and SAT -> AP 0.451, all of which were greater than zero. In addition, to further test the predictive relevance of the model, we obtained Stone–Geisser’s Q^2^ (the measure of cross-validated redundancy for all endogenous constructs) via the blindfolding algorithm of SmartPLS [91], which is shown in Figure 2. All Q^2^ values were found to be greater than 0, indicating that constructs had predictive relevance [89]. Finally, to test our research hypotheses regarding the significance of the paths, we obtained the standardized path coefficient (β) values and the coefficients of determination (R^2^) of the endogenous constructs in the research model; see Figure 2. 

## 6. Discussion

The use of SM platforms has become a key part of education, and it has grown increasingly significant in both course delivery and course evaluations. The work by Stathopoulou et al. showed a beneficial effect of the integration of SM in education on the profound learning experience of students [29]. SM can be used as a tool to support students and help instructors during their learning processes. Research has illustrated that the significant role of using SM platforms in the concepts of OL can be observed globally because these technologies increase learning, cooperation, and information sharing among students, teachers, and subject professionals as they are crucial for learning and training. The authors of this paper aimed to examine the real motives behind the use of SM in an international medical student community. We proposed a conceptual model that utilizes TAM and SCT. According to Rauniar, Rawski, Yang, and Johnson, the use of SM platforms to promote interpersonal interactions, communication, entertainment, and social bonding among users has become a global phenomenon [92]. In the context of OL, our results also provide important contributions to SCT and TAM [93]. Thus, we recommend the use of SM platforms for OL in higher education because they provide opportunities to students for interaction, ENG, and collaboration with peers, all of which improve their AP. Over time, we hope that many advisors will integrate SM platforms into educational programs in order to aid modern students and encourage OL [4,94]. The use of the most well-liked SM platform applications, such as WeChat, Weibo, Tencent Meeting, Twitter, Facebook, WhatsApp, and Google Classroom, for online class sessions is becoming more functional. Simultaneously, the widespread use of technology such as laptops, mobiles, and tablets (which allow for easy access to SM) can enhance students’ educational activities.

The present study has revealed that SM platforms aid the creation of learning environments by enhancing student cooperation, communication, and articulation. The findings of the study show that there is a significant and positive relationship between H1, H2, and H3 with OL. Most of the students reported that using SM platforms for OL is a good idea. In other words, the use of social media affects OL, which, in turn, has a significant impact on students’ AP through information sharing, material exchange, and peer discussion. When students engage in OL and enjoy using SM platforms, they also participate in discussions with subject specialists and peers while engaging with their own social presence. These findings are in line with those of earlier studies [11,13], which support the idea that SM platforms are useful for OL. We also identified that student collaboration could be promoted via the use of SM platforms in learning and teaching; consequently, adequate learning results and student AP can be increased through interactions with virtual communities. Similar results were also reported by Tarantino, McDonough, and Hua [95]. Another study showed that recently created apps have inspired students to utilize SM to learn in diverse educational environments [71]. Though SM has larger implications for classroom students, scholars have also investigated SM for use by technicians [96,97]. In her case study on technology, Bernadette Longo said that SM is an important element of the broad and complex social networks that comprise human technology [98].

OL was found to show significant relationships with H4 and H5. Through SM, OL improves the AP of students by enhancing the communication skills and knowledge exchange among fellow learners. Our analysis also indicated an essential correlation between OL and AP because students reported having confidence in improving their learning outcomes with greater accomplishments, greater productivity, and lower research workloads by using social media, and they expect to use it in the future. We believe that incorporating SM platforms into traditional blogging could positively increase the academic outcomes of students. Furthermore, according to the results of this study, regarding H6, the use of SM platforms can contribute to the creation of a supportive and learning-conducive atmosphere, which is invaluable for student ENG, student learning, lecturer teaching experiences, and academic supervision. SM can improve learning settings by encouraging interaction and ENG among students, as well as promoting team discussions and the completion of projects. Overall, this study and previous studies have shown that students may use SM platforms for engagement to increase their AP [65]. Related to this result, Balakrishnan and Gan reported that SM platforms could change educational methods and provide space for students to directly communicate and collaborate with different people around the world [71]. This idea is supported via two theoretical perspectives: SCT and computer-mediated learning (CML). SCT’s main emphasis is on social contact and collaboration, and CML advocates the omnipresent stresses of topographical hurdles. Hence, to gain useful learning experiences related to OL, it is necessary to develop social groups to apply and use OL abilities via SM platforms.

## 7. Conclusions

This research contributes to the field of knowledge on the student adoption of SM platforms for the benefit of OL; it also emphasizes the role of SM in the worldwide adoption of collaborative working and OL principles. Such resources are beneficial to studying and teaching because they help students understand, collaborate, and share knowledge. These conclusions were reached by developing and empirically evaluating a conceptual framework based on the TAM and SCT. The applications of internet resources and SM platforms as sources of learning are enormously important and essential for students and scholars. Our findings revealed that studying in a group of peers is advantageous to researchers and students because it can enhance group output. In this manner, students can efficiently propose new ideas and sentiments in group debates and collaborations with each other. Furthermore, using SM platforms for OL and ENG can enrich students’ learning experiences while facilitating team discussions. This study has shown that the PEU, PU, and IP of SM platforms positively effects students’ OL, ENG, and, ultimately, AP. Particularly in a time of growing focus on expediently delivering coursework through digital technologies, students, higher educational institutions, and policymakers may see positive impacts of SM platform adoption by students on OL. However, this research had limitations, such as its sample size of N = 233 and its focus on international medical students in Jiangsu province universities of China, both of which make it difficult to draw conclusive inferences about the conceptual model’s effectiveness; therefore, the replication of this study in other countries with different economic and cultural conditions is crucial.

### 7.1. Implications

The present research has a few significant implications for students, higher educational institutes, and policymakers. Understanding the link between the use of SM platforms and their beneficial impacts on student performance is critical to comprehend the function of SM during their studies. The findings may be useful for those who are interested in improving online learning or using SM platforms to facilitate OL. This research endorses the idea that students should be welcomed, rather than forced, by their learning instituations to make use of SM to achieve OL in order to improve their AP in higher education. Additionally, lecturers and supervisors must help students with any questions they might have regarding the use of SM or information sharing. Students’ knowledge-seeking experiences and research expertise can be enhanced through the provision of useful knowledge by lecturers and supervisors. Following our results, interventions to stop or at least diminish cyberstalking and bullying should be adopted by legislators in universities to avoid their detrimental impacts on student academic achievement. These measures may lead to the development of a blueprint for recognising the variables that are expected to have significant impacts on the use of SM platforms for open learning to improve AP. The authors of this study implemented a variety of theoretical and empirical analyses, but the ideas of the research emerged from practice and will serve as the foundation for the implementation of new theories and approaches in the framework of China’s adoption of OL. This could be the first time that SCT and TAM have been applied to higher education in China, specifically to investigate the effect of SM platforms on OL and student AP, and our findings showed that SCT, when combined with the TAM, was an important theory for examining the impact of SM use on students’ OL and AP in Chinese higher education. 

### 7.2. Future Perspective and Limitations 

Further research can be conducted to fill in the gaps caused by the limitations of this study. This research was quantitative; data were collected with online survey questionnaires and were cross-sectional. The sample size was small and only included international medical students studying in universities located in the Jiangsu province of China. Results could be different in other provinces or geographical locations, even in the same country. In this research, AP was collected through participants’ self-reported construct, which may add limitations to the outcome. Thus, future studies may consider including students’ grades as students’ actual reported performance and achievement. For this research, we used specific social networks such as WeChat, QQ, Tencent Meeting, and Weibo; however, future studies can consider other social networks sites such as Facebook, LinkedIn, Twitter, and blogs. Furthermore, in future studies, mixed research approaches can be used, and the model could be expanded to include other variables such as enjoyment, satisfaction, interactions with teachers, and knowledge sharing. 

## Figures and Tables

**Table 1:** Respondents’ demographic information.

Items		Percentage
Gender	Male	55.4
	Female	44.6
Education	Under Graduate	69.95
	Masters	21.03
	Doctoral	9.01
Social Media Use Frequency for Educational Purposes	2 h	10.1
	Almost 3 h	15.5
	Almost 5 h	27.9
	More than 5 h	46.5
	Pakistan	38.19
	Bangladesh	16.73
International Medical Student Home Country	India	24.2
	Malaysia	11.58
	Afghanistan	9.3

**Table 2:** Factor loadings, Cronbach’s alpha, rho_A, CR, AVE, and VIF.

Constructs	PU	PEU	IP	OL	ENG	AP	Cronbach’s a	rho_A	CR	AVE	VIF
PU							0.878	0.883	0.916	0.732	
PU1	0.817										1.806
PU2	0.836										2.184
PU3	0.867										2.491
PU4	0.901										2.836
PEU							0.833	0.865	0.892	0.680	
PEU1		0.832									1.232
PEU2		0.904									3.004
PEU3		0.873									2.524
PEU4		0.897									3.532
IP							0.871	0.876	0.912	0.722	
IP1			0.848								2.096
IP2			0.801								1.847
IP3			0.902								2.963
IP4			0.847								2.184
OL							0.896	0.896	0.928	0.762	
OL1				0.829							2.017
OL2				0.881							2.810
OL3				0.887							2.662
OL4				0.894							3.008
ENG							0.888	0.889	0.922	0.748	
ENG1					0.854						2.438
ENG2					0.870						2.632
ENG3					0.882						2.626
ENG4					0.853						2.364
AP							0.900	0.903	0.930	0.770	
AP1						0.859					2.245
AP2						0.899					3.060
AP3						0.878					2.796
AP4						0.872					2.439

**Table 3:** Discriminant validity.

Constructs	PU	PEU	IP	OL	ENG	AP
PU	**0.855**	*0.867*	*0.845*	*0.883*	*0.886*	*0.773*
PEU	0.433	**0.824**	*0.857*	*0.853*	*0.841*	*0.826*
IP	0.521	0.607	**0.849**	*0.873*	*0.821*	*0.786*
OL	0.489	0.547	0.623	**0.873**	*0.858*	*0.650*
ENG	0.333	0.631	0.573	0.577	**0.864**	*0.814*
AP	0.525	0.589	0.625	0.596	0.669	**0.877**

Note: Diagonal elements in bold represent Fornell and Larcker criteria, and those in italics represent heterotrait–monotrait (HTMT).

## Data Availability

The data analyzed in this study are available from the corresponding author on reasonable request: naeem@smail.nju.edu.cn.

## Appendix A

Questionnaire

Perceived Usefulness

PU1: Using social media for open learning can help me to make my learning more efficient.

PU2: Using social media for open learning can be helpful for my learning needs.

PU3: Using social media for open learning can increase my assignment productivity.

PU4: Using social media for open learning allows me to communicate with more people in short periods.

Perceived Ease of Use

PEU1: Using social media for open learning enables flexible interactions with others.

PEU2: I find it easy to use social media to do what I want to do.

PEU3: It is easy to become skillful at using social media.

PEU4: I find social media easy to use for open learning.

Interaction with peers

IP1: Social media facilitates interactions with my peers.

IP2: Social media gives me the opportunity to engage in discussion with my peers.

IP3: Social media allows for the exchange of information with my peers.

IP4: Social media facilitates dialogue with my peers.

Open learning

OL1: Open learning builds strong and engaging connections between students and tutors.

OL2: Open learning offers opportunity for interaction and communication with instructors, other students, and content experts.

OL3: Open learning provides opportunities to students for team cooperation (collaboration), which has a direct impact on their performance.

OL4: Students have a positive attitude toward the use of social media for open learning and academic purposes.

Engagement

ENG1: By using social media, I engage in interactions with my peers.

ENG2: By using social media, I engage in interactions with my lecturers.

ENG3: By using social media, I have learned how to work with others effectively.

ENG4: By using social media, I have become satisfied with my engagement with studies.

Academic Performance

AP1: Social media has led to a better learning experience in this module.

AP2: Social media has allowed me to better understand my studies.

AP3: Social media is helpful in my studies and makes it easy to learn.

AP4: Social media improves my academic performance.
